# Terson syndrome with no cerebral hemorrhage: A case report

**DOI:** 10.3892/etm.2013.1400

**Published:** 2013-11-11

**Authors:** XIAO-BIN JING, LI-QIAN SUN

**Affiliations:** 1 Department of Ophthalmology, Tangshan Ophthalmology Hospital, Tangshan, Hebei 063000, P.R. China; 2 Department of Neurosurgery, Tangshan Gongren Hospital, Tangshan, Hebei 063000, P.R. China

**Keywords:** cerebral hemorrhage, intracranial pressure, traumatic brain injury, Terson syndrome, vitreous hemorrhage

## Abstract

The present study reports the case of a 33-year-old male who presented with Terson syndrome with no cerebral hemorrhage secondary to traumatic brain injury (TBI). A computed tomography scan of the patient, who had sustained an impact injury to the right occipital region, showed no cerebral lesion. Ophthalmoscopy clearly demonstrated vitreous hemorrhage in both eye globes. Vitreous hemorrhage, which results from an abrupt increase in intracranial pressure (ICP), is associated with TBI. In this case, the visual disturbance was attributed to Terson syndrome secondary to TBI. Therefore, close ophthalmological and radiological evaluation is required in patients with TBI, in order to enable the diagnosis of Terson syndrome and an early vitrectomy.

## Introduction

Terson syndrome was originally defined as a vitreous hemorrhage arising from an aneurysmal subarachnoid hemorrhage (SAH) (1); however, this definition has since been expanded to include retinal and preretinal hemorrhages (2,3). An abrupt increase in intracranial pressure (ICP) is typically responsible for this type of intraocular hemorrhage (4). Terson syndrome, which occurs in 10–20% of patients with SAH (5,6), may be caused by a severe cerebral hemorrhage induced by a head injury or by a subdural or epidural hematoma; however, to the best of our knowledge, there have been no studies describing patients with Terson syndrome who have not presented with cerebral hemorrhage following traumatic brain injury (TBI) (7).

The presence of vitreous and preretinal hemorrhage without cerebral hemorrhage occurring secondary to TBI, creates challenges for oculists and neurosurgeons and reduces the likelihood of Terson syndrome being considered. This study presents a rare case of Terson syndrome with no cerebral hemorrhage secondary to TBI.

## Case report

**Figure 1:**
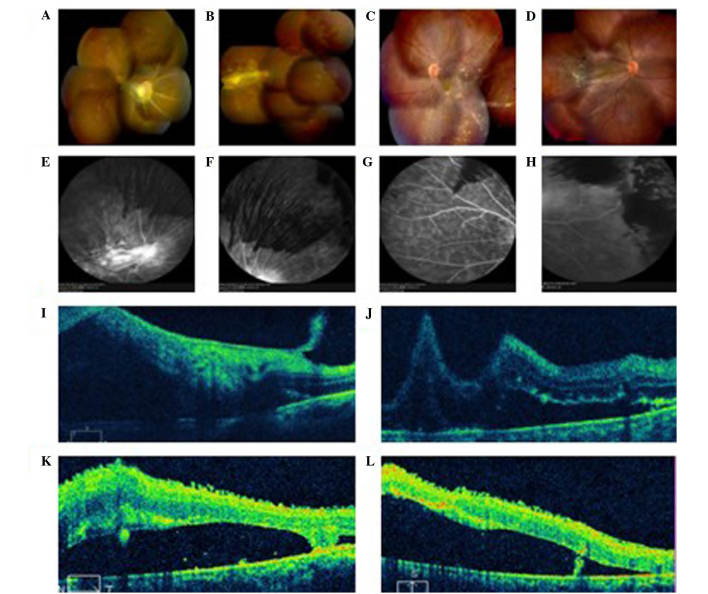
Ocular fundus images from a patient with Terson syndrome with no cerebral hemorrhage. Color fundus photography showed peripheral preretinal hemorrhage, macula lutea and optic papilla traction in the patient’s (A) right and (B) left eyes prior to vitrectomy. Two months subsequent to the vitrectomy, the retinal traction was released in the patient’s (C) right and (D) left eyes. Prior to the vitrectomy, fundus fluorescence angiography (Spectralis HRA; Heidelberg Engineering GmbH, Heidelberg, Germany) showed that the vitreous hemorrhage blocked the fluorescence in the peripheral retina of the patient’s (E and F) right and (G and H) left eyes. In addition, optical coherence tomography showed retinal detachment, macula lutea and optic papilla traction in the (I) right and (J) left eyes. Two months subsequent to the vitrectomy, optical coherence tomography showed retinal nerve fiber layer detachment in the macula lutea in the patient’s (K) right and (L) left eyes.

The patient, a 33-year-old male miner, was hit in the right occipital region by a boulder while mining, three hours prior to admission to a local clinic. Computed tomography showed no cerebral lesion. One week later, the patient presented with sudden visual disturbance. Ophthalmoscopic examination detected vitreous hemorrhage in both eyes and the vitreous hemorrhage was subsequently managed with conservative treatment. The patient was referred to Tangshan Ophthalmology Hospital (Tangshan, China) due to visual deterioration six months subsequent to the onset of the symptoms. Visual acuity was 20/400 in each eye. The patient’s ophthalmic signs were as follows: Peripheral vitreous hemorrhage, peripheral preretinal hemorrhage, macula lutea and optic papilla traction and tractional retinal detachment (Fig. 1A, B and E–J). As a result, the patient was diagnosed with Terson syndrome and a vitrectomy was conducted. Two months subsequent to the surgery, the visual acuity was measured to be 20/1,000 in each eye. Color fundus photography, using a nonmyd 7™ camera (Kowa Company Ltd, Nagoya, Japan), showed that the retinal traction was released. In addition, optical coherence tomography, using a Stratus OCT 3,000 tomography instrument (Carl Zeiss Meditec Inc, Dublin, CA, USA), demonstrated retinal nerve fiber layer detachment in the macula lutea (Fig. 1C, D, K and L). Informed consent was obtained from the patient for the procedure and publication of the study.

## Discussion

Terson syndrome, a known complication of cerebral hemorrhage, may lead to loss of vision. Vision loss develops subsequent to cerebral hemorrhage, as a result of vitreous hemorrhage caused by retinal capillary disruption. The pathogenesis of Terson syndrome has been suggested to be associated with an impairment of the retinal microcirculation as a result of increased ICP following cerebral hemorrhage, which ultimately results in venous hypertension and hemorrhage (8). Therefore, the occurrence of the syndrome is closely correlated with poor outcome in terms of sequela from increased ICP.

The present case showed that the absence of cerebral hemorrhage secondary to TBI may induce an abrupt increase in ICP, resulting in Terson syndrome. Hoving *et al*(9) described a patient with an obstructive hydrocephalus, which was treated endoscopically; however, due to the inadvertent use of a pressure bag during rinsing, in combination with a blocked outflow channel, a steep rise in ICP occurred. Postoperatively, the patient experienced visual disturbances caused by bilateral retinal hemorrhage, and, ultimately, an iatrogenic Terson syndrome was diagnosed (9). In the present case, it is hypothesized that the cause of the Terson syndrome may have been a rapid elevation in ICP at the time of TBI, which affected peripapillary structures through the intervaginal space of the optic nerve sheath (10).

The immediate examination of patients with TBI for intraocular hemorrhage is important due to the fact that severe, non-clearing vitreous hemorrhage may result in visual loss. A prompt diagnosis of Terson syndrome for patients with vitreous hemorrhage suffering from TBI with no cerebral hemorrhage is required to enable vitrectomy to be performed earlier.

## Abbreviations

ICP: intracranial pressure
SAH: subarachnoid hemorrhage
TBI: traumatic brain injury
